# oXiris-Based Blood Purification Efficacy in Adults with Septic Shock and Acute Kidney Injury: The PURE Multicenter Pilot Randomized Clinical Trial

**DOI:** 10.3390/jcm15208028

**Published:** 2026-10-16

**Authors:** Ahmed Mady, Waleed Tharwat Aletreby, Khaled Sewify, Fahad Ali Faqihi, Rayan AlShayaa, Khaddash N. Issa, Saleh Almahwi, Jennifer Q. Gano, Adel Ali Alkenani, Ahmed Shaaban, Mohammad Al Odat, Abdullah Alquaydheb, Abdulrahman Alanazi, Basel Almuabbadi, Muhammad Asim Rana, Basheer Abdelrahman, Huda Mhawish, Amr El-Husseini, Marcus Ewert Broman

**Affiliations:** 1 Critical Care Department, King Saud Medical City, Riyadh 11196, Saudi Arabia; afmady@hotmail.com (A.M.); ahmadwaleed2005@gmail.com (W.T.A.); dr.faqihi677@gmail.com (F.A.F.); khaddash@rfhc.gov.sa (K.N.I.); jen.quilongano@gmail.com (J.Q.G.); adalkenani@moh.gov.sa (A.A.A.); abdullah.quayhdeb@gmail.com (A.A.); dr.abdulrahman.med@gmail.com (A.A.); b.almuabbadi@rfhc.gov.sa (B.A.);; 2 Department of Anesthesiology and Intensive Care, Faculty of Medicine, Tanta University, Tanta 31527, Egypt; 3 Critical Care Department, King Fahd Military Medical Complex, Dhahran 31932, Saudi Arabia; ahmedshaabanonline@gmail.com; 4 Critical Care Department, Amna Inayat Medical College, Lahore 54000, Pakistan; drasimrana@yahoo.com; 5 Critical Care Department, King Abdullah Specialized Hospital, National Guard Health Affairs, Buraidah 52569, Saudi Arabia; 6 Division of Nephrology, Bone and Mineral Metabolism, University of Kentucky, Lexington, KY 40506, USA; amr.elhusseini@wiccem.org; 7 Perioperative and Intensive Care, Skåne University Hospital, 222 42 Lund, Sweden; marcus.broman@med.lu.se

**Keywords:** oXiris, septic shock, acute kidney injury, continuous renal replacement therapy, continuous venovenous hemodialysis, blood purification, randomized clinical trial

## Abstract

**Background/Objectives:** Randomized evidence for the oXiris adsorptive membrane in septic shock remains limited. We estimated its effect on 28-day mortality and explored short-term physiologic outcomes in adults with septic shock and acute kidney injury requiring continuous renal replacement therapy. **Methods:** PURE was a multicenter, single-blind, parallel-group pilot randomized trial in Saudi Arabia. Participants were randomized 1:1 to oXiris-based or conventional continuous venovenous hemodialysis. The primary comparison used unadjusted mortality risk estimates. Secondary observations were summarized descriptively; adjusted and post-hoc Bayesian sensitivity analyses are reported in the main text and supplement. **Results:** Of 204 randomized participants, 202 were analyzed (oXiris, 100; control, 102). Twenty-eight-day mortality was 39.0% (39/100) versus 47.1% (48/102), respectively (risk ratio, 0.83; 95% CI, 0.60–1.14; risk difference, −8.1 percentage points; 95% CI, −21.7 to 5.6; *p* = 0.247). Reported 72 h norepinephrine dose, lactate, C-reactive protein, and Sequential Organ Failure Assessment (SOFA) score were lower with oXiris; reported intensive care unit (ICU) stay was also shorter. Secondary descriptive observations are limited by unverifiable time-specific denominators and handling of intercurrent events. Premature filter clotting was reported in 8/100 versus 7/102 participants; no device-related adverse events were reported, but safety ascertainment was limited. **Conclusions:** This pilot trial did not demonstrate a statistically significant reduction in 28-day mortality, although mortality was numerically lower in the oXiris group. Consistent protocol implementation and encouraging, hypothesis-generating physiologic observations provide a foundation for a definitive trial with complete longitudinal ascertainment.

## 1. Introduction

Sepsis is defined as life-threatening organ dysfunction caused by a dysregulated host response to infection and is a major cause of critical illness [1,2]. Septic shock is characterized by vasopressor-dependent hypotension and elevated lactate despite adequate resuscitation and is associated with substantial mortality [1,2]. Acute kidney injury (AKI) frequently complicates septic shock and may necessitate continuous renal replacement therapy (CRRT) [3].

The pathophysiology of sepsis includes dysregulated responses to microbial products, including lipopolysaccharide (endotoxin), and to pro-inflammatory and anti-inflammatory mediators [4]. Extracorporeal blood purification has therefore been investigated as an adjunct to antibiotics and supportive care [5,6]. Adsorptive approaches are not interchangeable: surface-treated AN69 (AN69ST) membranes are used within CRRT, whereas CytoSorb and polymyxin B devices provide distinct hemoadsorption strategies [7,8,9,10].

The oXiris membrane is an AN69-based CRRT membrane with additional adsorptive surface modifications: an AN69 base polymer with cytokine-binding properties, a polyethyleneimine coating intended to enhance endotoxin adsorption, and surface heparin grafting [7,10]. Mechanistic studies have described endotoxin and cytokine removal, but this does not establish improved patient outcomes [10]. Conventional AN69-based membranes can also have adsorptive properties; because the control models in PURE were not centrally documented, the comparator should not be assumed to have been nonadsorptive.

Small observational studies and mechanistic reports have described improvements in hemodynamic parameters and vasopressor requirements during oXiris treatment, but randomized evidence remains limited [7,10]. Two systematic reviews reported favorable mortality and organ-function signals while emphasizing methodological limitations and low certainty [11,12].

The PURE trial evaluated oXiris-based continuous venovenous hemodialysis (CVVHD), compared with conventional CVVHD, in ICU adults with septic shock and AKI. Its primary objective was to estimate the treatment effect on 28-day all-cause mortality. Secondary objectives explored short-term hemodynamic, inflammatory, organ-function, and safety observations. CRRT is used below as the overarching modality, CVVHD as the specific technique delivered, and blood purification as the broader therapeutic concept.

## 2. Materials and Methods

### 2.1. Study Design and Setting

PURE was an investigator-initiated, multicenter, single-blind, two-group, parallel pilot randomized clinical trial conducted at tertiary ICUs: King Saud Medical City and a collaborating center in Saudi Arabia. Recruitment occurred from 21 January through 13 November 2025, and stopped after the feasibility target had been reached. The trial used an exploratory superiority framework with 1:1 allocation. This report follows CONSORT 2025 and the CONSORT extension for randomized pilot and feasibility trials [13,14].

### 2.2. Patient and Public Involvement

Patients and members of the public were not involved in the design, conduct, analysis, or reporting of the trial. Participants or legally authorized representatives provided consent as described below.

### 2.3. Ethics, Registration, and Protocol Access

The study was conducted in accordance with the Declaration of Helsinki and approved by the Institutional Review Board of King Saud Medical City (reference H1RI-24-Jul22-01; approval memorandum dated 4 September 2022). The consecutive continuing approvals collectively covered 16 January 2025, to 15 January 2026; the individual validity periods are reported in the Institutional Review Board Statement. Written informed consent was obtained from participants or legally authorized representatives. An emergency-consent pathway permitted enrollment before a representative was available, with subsequent consent required for continued data use. Good Clinical Practice principles were followed.

PURE was prospectively registered with Research Registry on 22 December 2024 (researchregistry10932; https://www.researchregistry.com/) before first enrollment on 21 January 2025. The original entry described a single-center trial; recruitment subsequently included a collaborating ICU. The randomized treatment comparison and primary 28-day mortality endpoint remained unchanged. A dated registry update documenting the additional center before recruitment ended was not available for verification. Institutional continuing approval and the public registry amendment history are reported separately. The protocol, case-report form, and archived statistical documentation are available from the corresponding author, subject to institutional approval.

### 2.4. Participants

Adults were eligible if they were aged 18 years or older, met Sepsis-3 criteria for septic shock, had AKI defined by Kidney Disease: Improving Global Outcomes criteria, and had an independent clinical decision by the treating intensivist to initiate CRRT. Exclusion criteria were age younger than 18 years; absence of septic shock; CRRT for a reason other than AKI, including chronic dialysis; pregnancy or lactation; do-not-resuscitate or end-of-life designation; heparin allergy or heparin-induced thrombocytopenia type II; shock of non-septic origin; decompensated cirrhosis; metastatic or hematologic malignancy; intracranial hemorrhage or uncontrolled coagulopathy; previous organ transplantation; HIV infection; or active tuberculosis.

### 2.5. Randomization and Masking

Successive eligible participants at all participating centers were randomized individually in a 1:1 ratio using central telephone allocation with blocks of four and no center stratification. Allocation was concealed until eligibility had been confirmed. The central, unstratified allocation procedure established for the original single-center design was retained when recruitment expanded. Treating clinicians could not be masked because circuit preparation differed between groups. Participants or their surrogates were masked to assignment, and the statistical analyst received coded group labels. These allocation and masking procedures were applied consistently across participating centers. The identity of the sequence generator and the random-number software were not specified in the available trial documentation.

### 2.6. Interventions and Co-Interventions

The same core CVVHD protocol and Prismaflex platform were used in both treatment groups at all participating centers. All participants were initiated on CRRT in CVVHD mode, with a target prescribed effluent dose of 20–30 mL/kg/h and unfractionated heparin administered through the extracorporeal circuit. Vascular access was obtained through a double-lumen 12 Fr catheter in the internal jugular or femoral vein. In the intervention group, the same oXiris membrane was used across centers; blood flow was maintained at 100–200 mL/min, and the filter was replaced every 24 h for up to 72 h while treatment continued. The control group received CVVHD using conventional Prismaflex-compatible CRRT membranes according to local practice, with the same prescribed dose. The control-filter model, control replacement schedule, actual delivered dose, and circuit downtime were not captured centrally. Table S1 separates the common protocol elements from the unavailable treatment-delivery measurements. oXiris filters were supplied in kind by the manufacturer, which had no role in trial design, conduct, data collection, analysis, interpretation, manuscript preparation, or the decision to submit.

Both groups at all participating centers received standard sepsis care. Antimicrobials, source control, fluid therapy, corticosteroids, ventilation, and vasopressor titration were managed by the treating teams and were not otherwise protocolized. The treating intensivist determined when CRRT was discontinued. No study-specific pharmacokinetic assessment of antibiotic or sedative adsorption was documented.

### 2.7. Outcomes and Safety Assessment

The primary endpoint was 28-day all-cause mortality, with day 1 defined as the day of CRRT initiation. Day 28 vital status was available for all 202 participants included in the modified intention-to-treat cohort.

Exploratory secondary outcomes included norepinephrine dose, serum lactate, mean arterial pressure, Sequential Organ Failure Assessment (SOFA) score, serum creatinine, PaO_2_/FiO_2_ ratio, C-reactive protein (CRP), and white blood cell count at 72 h; ICU and hospital length of stay; and time to vasopressor discontinuation. Length of stay was not a 72 h measurement. Protocol-listed subgroup variables were sex, age, Acute Physiology and Chronic Health Evaluation (APACHE) IV score, baseline norepinephrine dose, and acute respiratory distress syndrome (ARDS) severity. Formal subgroup effect estimates and interaction tests are not reported. Premature filter clotting and device-related adverse events were investigator-reported, without independent adjudication or systematic surveillance for uncommon harms. The study records do not document protocolized antibiotic or sedative concentration monitoring, pre-/post-filter drug sampling, or pharmacokinetic assessment of adsorption.

### 2.8. Sample Size

No formal efficacy sample-size calculation was performed. The feasibility target was approximately 100 participants per group, based on expected recruitment and filter availability. The archived trial documentation did not specify an interim efficacy analysis or a formal statistical stopping boundary.

### 2.9. Statistical Analysis

Analyses used a modified intention-to-treat population, retaining participants in their assigned groups except for two oXiris participants whose permission for continued data use was withdrawn. Day 28 vital status was available for all 202 analyzed participants. Continuous variables are presented as mean (standard deviation), unless otherwise stated, and categorical variables as *n* (%). The unadjusted randomized comparison was primary. Baseline standardized differences are descriptive measures of imbalance, with signed differences expressed as oXiris minus conventional CVVHD; they are not used as significance tests or to validate randomization [14]. Secondary outcomes are presented as descriptive group summaries; their original analysis populations and intercurrent-event rules could not be verified. A full participant-level intention-to-treat analysis including all 204 participants was not performed. A post-hoc aggregate sensitivity calculation considered three hypothetical outcomes for the two consent withdrawals: neither, one, or both participants dying by day 28. These scenarios do not use withdrawn records or represent observed outcomes (Note S5; Table S13).

The primary outcome was summarized using the risk ratio and absolute risk difference with 95% confidence intervals; the two-sided Pearson chi-square *p*-value is also reported. The archived covariate-adjusted logistic-regression sensitivity model included treatment group, age, mean arterial pressure, PaO_2_/FiO_2_ ratio, lactate, serum creatinine, platelet count, C-reactive protein, white blood cell count, and APACHE IV score. This 10-predictor model was an exploratory sensitivity analysis and did not replace the primary unadjusted randomized comparison. Isolated laboratory measurements in three control and two oXiris participants were reported as multiply imputed (Supplementary Note S1).

A post-hoc contextual Bayesian sensitivity analysis was undertaken to complement the frequentist mortality estimates by expressing the probability of lower mortality odds under explicit prior assumptions and examining sensitivity to the amount of external information used. It did not replace the primary randomized comparison or increase the trial’s statistical power. The analysis used the unadjusted 28-day mortality counts and an indirect Tigris-informed prior [9]. The reference scenario retained 75% of the prior precision. A normal approximation on the log-odds scale was used, with sensitivity analyses from 0% to 100% retained precision. The prior was centered at ln(0.80); its standard deviation was approximated from the reported interval width (approximately 0.3486). Supplementary Note S3 provides the formulas and a reproducible aggregate-data calculation. The analysis was added after Tigris became available and was therefore post-hoc, including the choice of reference weight; no prespecified Bayesian success threshold is applied. Only the unadjusted Bayesian calculation is retained; the reason for omitting an earlier adjusted Bayesian output is documented in Note S3.

The earlier vasopressor analyses included nominal 72 h measurements, trapezoidal area under the curve (AUC), and a longitudinal mixed-effects model. The retained norepinephrine means and AUC summaries are descriptive because the time-specific analysis population and intercurrent-event coding could not be established. Note S4 documents the reporting scope, and Section 4.2 discusses its implications. Original analyses used Stata Release 17 (StataCorp LLC, College Station, TX, USA).

Reported trial-baseline and 72 h group means are presented side by side in Table S14 (Note S6). These marginal summaries may describe different participants and are not an analysis of individual change. No arithmetic change columns, change-score confidence intervals, *p*-values, or baseline-adjusted participant-level analyses of these outcomes are reported. The available baseline is the trial baseline, not a separately documented ICU-admission assessment.

### 2.10. Use of Generative Artificial Intelligence

During preparation and revision of this manuscript, the authors used ChatGPT powered by OpenAI’s GPT-6 model to assist with language editing, document organization, and figure formatting. AI-assisted image-design tools were also used during development of the graphical abstract. No participant-level observations were generated, altered, or reconstructed using generative AI. Aggregate numerical checks do not constitute a reanalysis of participant-level data. The authors reviewed and edited the assisted content and remain responsible for the accuracy, interpretation, originality, and integrity of the manuscript and its supporting materials.

## 3. Results

### 3.1. Participant Flow and Recruitment

**Figure 1:**
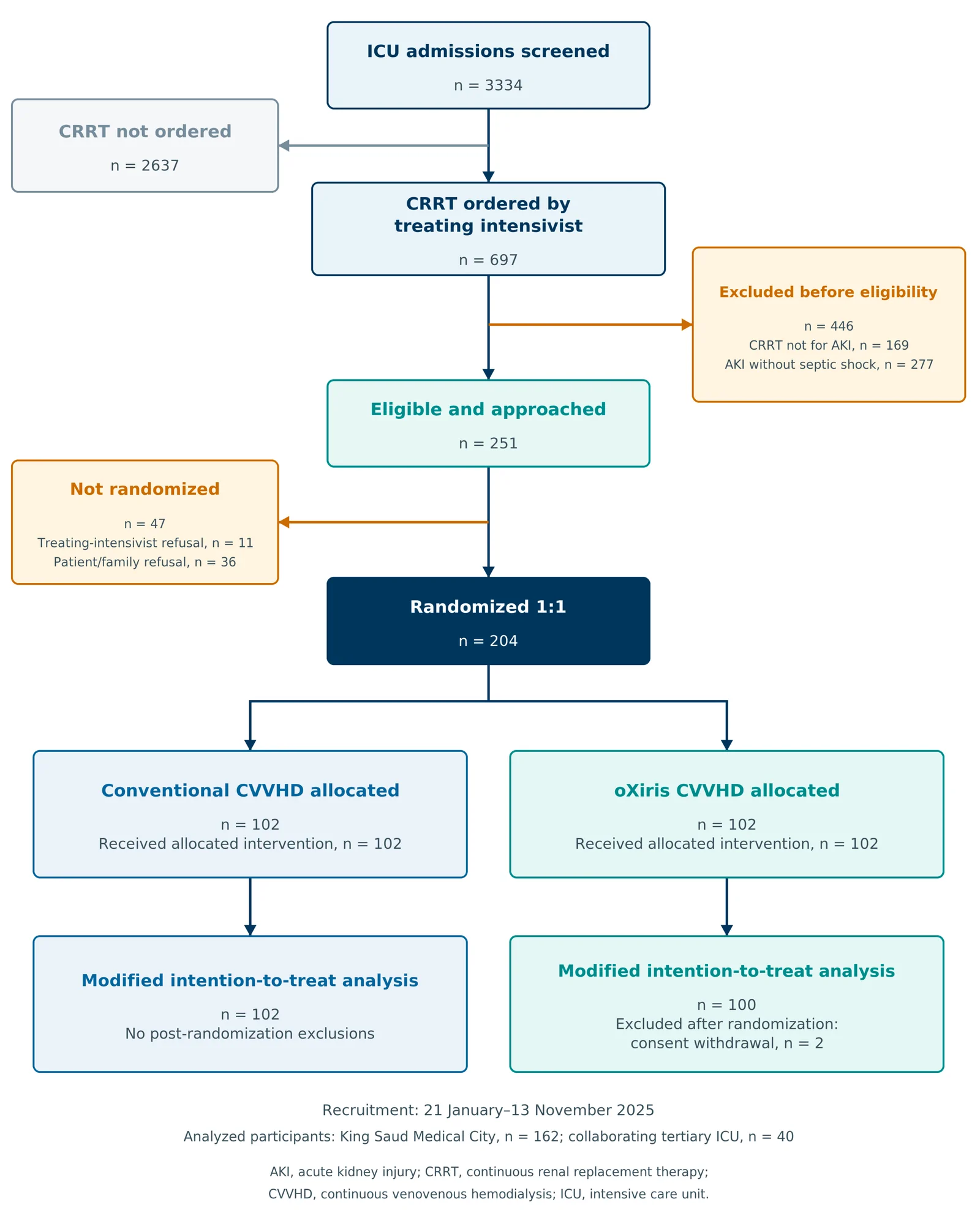
Participant flow in the PURE multicenter pilot randomized clinical trial. The modified intention-to-treat cohort excluded two participants randomized to oXiris after consent for continued data use was withdrawn. Arrows show participant progression or exclusion. The treatment-group boxes are blue for conventional CVVHD and teal for oXiris CVVHD; amber boxes show exclusions or non-randomization. AKI, acute kidney injury; CRRT, continuous renal replacement therapy; CVVHD, continuous venovenous hemodialysis.

During the recruitment period, 3334 ICU admissions were screened. CRRT was ordered for 697 patients; 446 were excluded because CRRT was not for AKI (*n* = 169) or because AKI occurred without septic shock (*n* = 277). Of 251 eligible patients, 47 were not randomized because the treating intensivist declined enrollment (*n* = 11) or the patient/family declined participation (*n* = 36). A total of 204 participants were randomized, 102 per group. Two participants assigned to oXiris were excluded after withdrawing consent for continued data use, leaving 100 participants in the oXiris group and 102 in the conventional CVVHD group in the modified intention-to-treat cohort (Figure 1). Of the analyzed cohort, 162 (80.2%) were enrolled at King Saud Medical City, and 40 (19.8%) at the collaborating tertiary ICU.

### 3.2. Baseline Characteristics

Table 1 summarizes baseline demographic and clinical characteristics. Absolute standardized differences were approximately 0.22 for mean arterial pressure, creatinine, and CRP; the largest displayed differences concerned PaO_2_/FiO_2_ (approximately 0.45) and baseline norepinephrine (0.30). These describe the magnitude of baseline differences, not statistical-significance thresholds.

### 3.3. CVVHD Administration and Safety

All analyzed participants received their allocated CVVHD strategy under the shared core protocol, using the Prismaflex platform in both groups at all participating centers. Both groups had a target prescribed effluent dose of 20–30 mL/kg/h and received unfractionated heparin for circuit anticoagulation. The oXiris protocol specified replacement every 24 h for up to 72 h at each center. Premature circuit/filter clotting within the first 72 h was reported in 8 of 100 oXiris participants and 7 of 102 conventional CVVHD participants. No events were reported under the separate category of device-related adverse events (Table S1). These safety observations were investigator-reported.

### 3.4. Source of Infection

Positive-culture rates were similar in the conventional CVVHD and oXiris groups (91.2% vs. 89.0%). Abdominal infection was the most frequently reported source (51.0% vs. 58.0%), followed by pulmonary infection (34.3% vs. 28.0%). Some participants had more than one culture source; descriptive source distributions are provided in Table S2.

### 3.5. Primary Outcome

**Figure 2:**
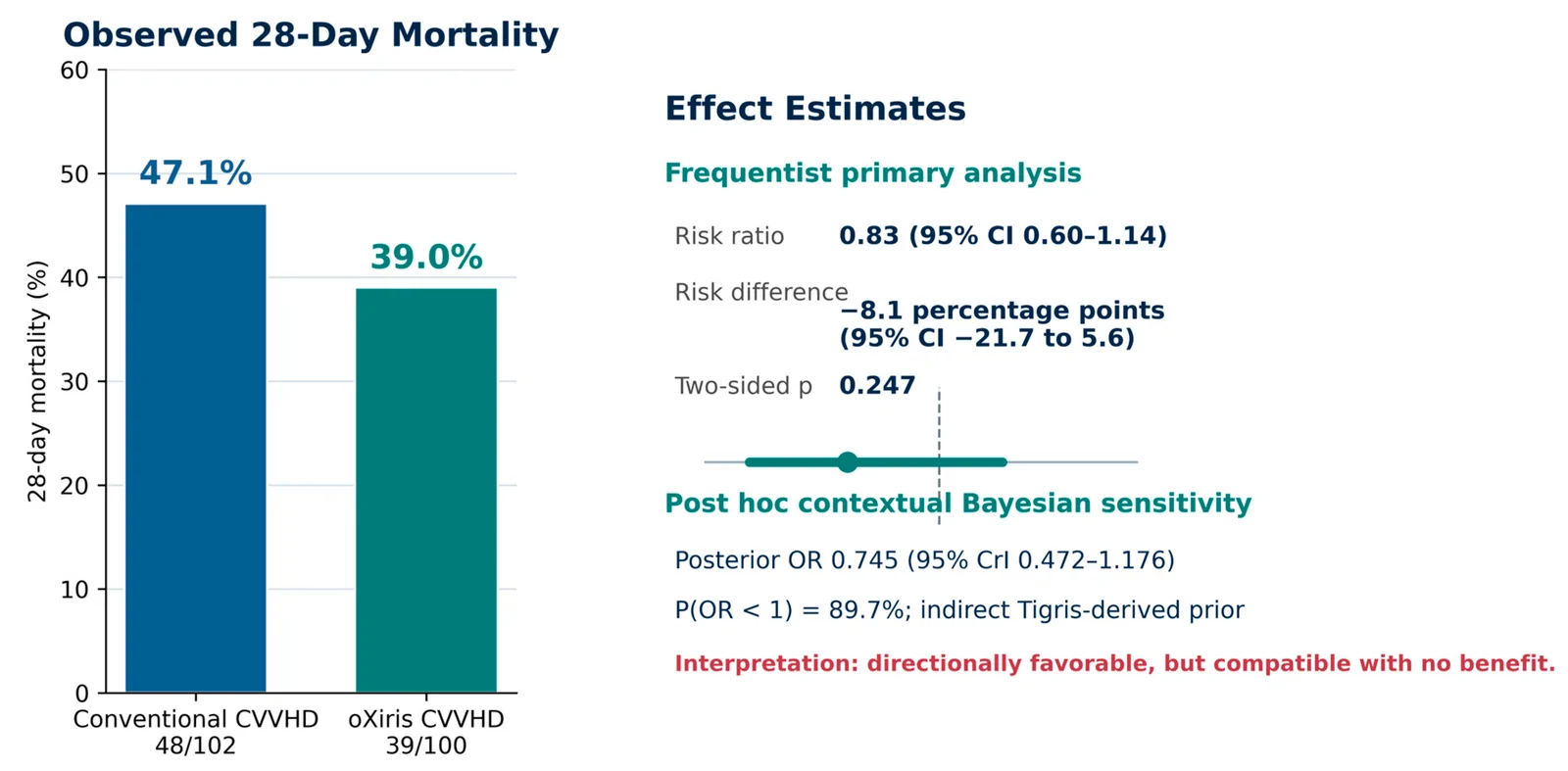
Primary outcome: 28-day all-cause mortality. The frequentist randomized comparison is primary. The Bayesian estimate is a post hoc contextual sensitivity analysis using a discounted indirect prior from Tigris. The dashed vertical line marks the null value of 1 (no difference between groups). CI, confidence interval; CrI, credible interval; OR, odds ratio.

By day 28, 39 of 100 participants (39.0%) in the oXiris group and 48 of 102 participants (47.1%) in the conventional CVVHD group had died. Comparing oXiris with conventional CVVHD, the risk ratio was 0.83 (95% CI, 0.60–1.14). The absolute risk difference, calculated as oXiris minus conventional CVVHD, was −8.1 percentage points (95% CI, −21.7 to 5.6); the two-sided Pearson chi-square *p*-value was 0.247 (Table 2; Figure 2). In the original covariate-adjusted sensitivity model, the adjusted odds ratio for oXiris was 0.610 (95% CI, 0.326–1.141; *p* = 0.122) (Table 3). Age and PaO_2_/FiO_2_ ratio were associated with mortality within that original exploratory model (Supplementary Tables S3–S7; Figures S1 and S2).

### 3.6. Bayesian Sensitivity Analyses

In the post-hoc unadjusted Bayesian reference analysis, the posterior odds ratio was 0.745 (95% CrI, 0.472–1.176), with an 89.7% posterior probability that the mortality odds ratio was below 1. Across 0–100% retained prior precision, this probability ranged from 87.6% to 90.3% (Tables S8 and S9; Figure S3). The calculation and reporting history are described in Note S3. These probabilities are conditional on the approximate, indirect prior and do not establish clinical efficacy.

### 3.7. Exploratory Secondary Outcomes

Reported mean ICU length of stay was 13.7 days with oXiris and 16.1 days with conventional CVVHD; reported mean hospital stay was 20.1 and 21.4 days, respectively.

At 72 h, reported mean norepinephrine was 0.18 vs. 0.25 µg/kg/min, lactate 2.2 versus 2.8 mmol/L, CRP 54.6 versus 75.2 mg/L, and SOFA score 3.1 vs. 3.5 in the oXiris and conventional CVVHD groups, respectively (Table 2). The values are retained as descriptive group summaries only; no inferential comparison is reported.

**Figure 3:**
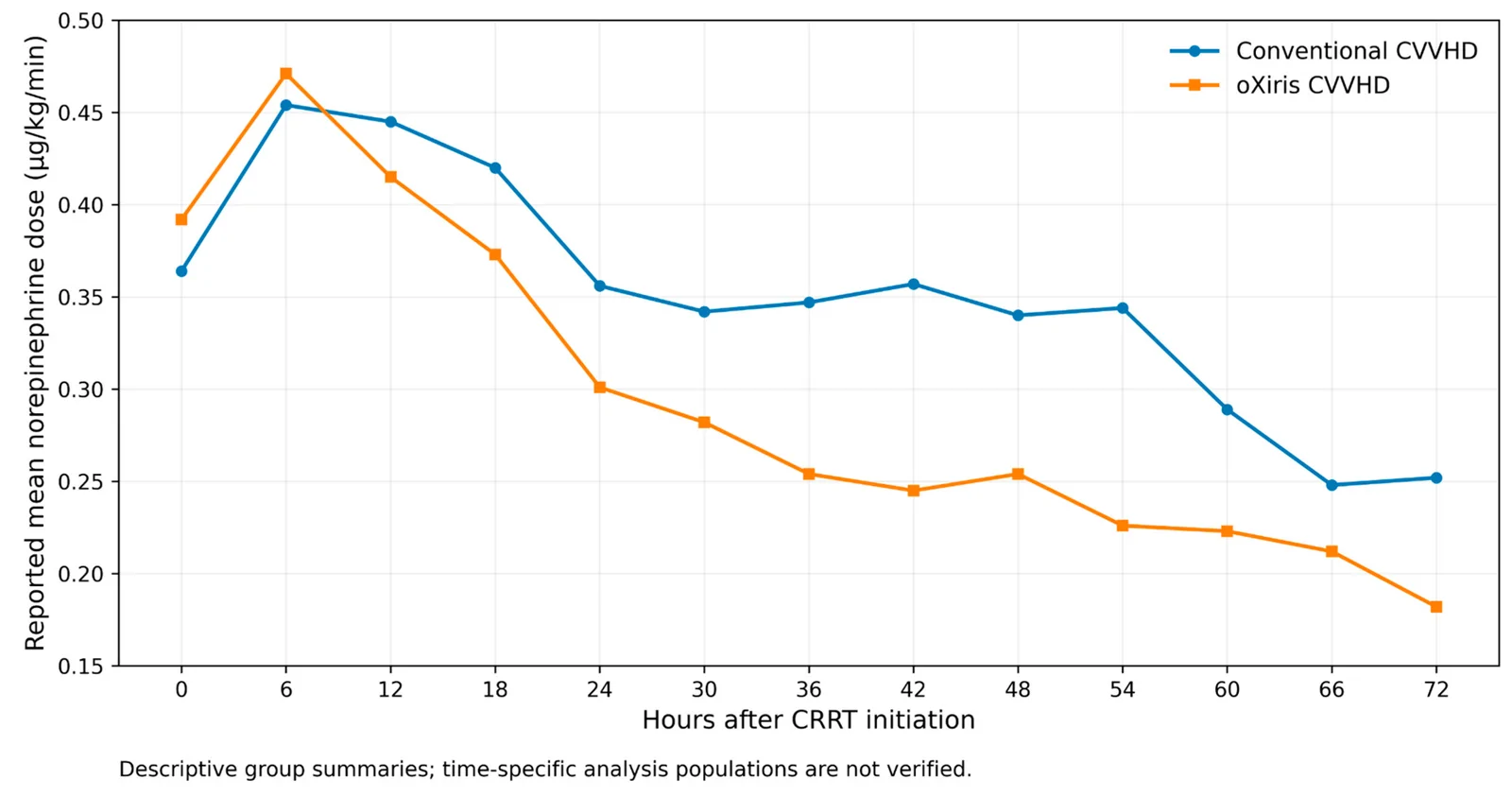
Reported mean norepinephrine dose through 72 h. The plotted values are descriptive group summaries, not a verified complete longitudinal cohort. Error bars are omitted because time-specific numbers assessed and intercurrent-event coding could not be verified. Detailed limitations are provided in Section 4.2 and Note S4. No new longitudinal analysis was performed.

The reported norepinephrine AUC means were 21.3 with oXiris and 25.5 with conventional CVVHD. The AUC summaries and mean trajectory are provided in Tables S10 and S11 and Figure 3.

Table S14 presents the available trial-baseline and 72 h means side by side. It does not estimate within-patient change, compare individual recovery between groups, or substitute for a paired analysis. Note S6 explains the scope of this descriptive display.

Under the three hypothetical aggregate scenarios for the two consent withdrawals, the mortality risk difference ranged from −8.8 to −6.9 percentage points; each 95% confidence interval included no effect. These bounds are sensitivity calculations, not observed outcomes from a full intention-to-treat analysis (Note S5; Table S13).

### 3.8. Subgroup Reporting

The protocol listed sex, age, APACHE IV score, baseline norepinephrine dose, and ARDS severity as subgroup variables. Table S12 summarizes the subgroup reporting decisions; Note S4 explains the revision of earlier subgroup wording. No treatment-responsive subgroup or selection threshold is established. The investigator has clarified the earlier age-stratum reporting error and supplied corrected counts that agree with the reported primary mortality totals. Subgroup treatment-effect estimates remain omitted, and the primary mortality totals are unchanged (Note S4; Table S12).

## 4. Discussion

In this multicenter pilot randomized trial, 28-day mortality was numerically lower with oXiris-based CVVHD than with conventional CVVHD, but the difference was not statistically significant. The point estimate favored oXiris, while the primary confidence interval remained compatible with clinically important benefit, no effect, or some harm. The original exploratory adjusted estimate and the post-hoc unadjusted Bayesian calculation did not resolve this uncertainty. Lower reported 72 h norepinephrine, lactate, CRP, and SOFA values and shorter reported ICU stay are encouraging signals for further investigation but remain descriptive and hypothesis-generating rather than evidence of a treatment effect on weaning, hemodynamic recovery, or organ recovery.

oXiris combines renal support with cytokine- and endotoxin-adsorptive surface properties. The AN69 copolymer contributes cytokine adsorption, the polyethyleneimine coating facilitates endotoxin binding, and surface heparin modifies membrane thrombogenicity [10,11,12,15]. These mechanisms provide biological plausibility but do not by themselves establish a patient-important clinical benefit.

The mortality result is consistent with an uncertain and heterogeneous literature. Small randomized studies and observational cohorts have reported favorable, neutral, and occasionally unfavorable point estimates [16,17,18,19,20,21,22,23,24,25,26]. Meta-analyses have suggested possible benefits but emphasized low certainty, methodological limitations, and the predominance of nonrandomized evidence [11,12]. PURE adds a randomized estimate in a clinically defined septic shock–AKI population, but its interval is too wide for a definitive efficacy conclusion.

The observed absolute risk difference of −8.1 percentage points may be clinically important, but its 95% confidence interval ranges from a 21.7-point reduction to a 5.6-point increase. Because the interval includes no benefit and possible harm, a single observed number needed to treat would be unstable and is not presented [27]. The post-hoc Bayesian calculation provides conditional context under an indirect prior; it does not resolve the uncertainty in the primary randomized comparison.

Several reports provide biologic and clinical context for the 72 h observations [10,19,22,23,24,25,26]. PURE adds descriptive observations under a shared core CVVHD protocol; the limitations of longitudinal ascertainment and attribution of these findings specifically to the membrane are brought together in Section 4.2.

The 2024 Asia–Pacific expert consensus proposed considering oXiris in selected CRRT-treated patients with marked hemodynamic instability or hyperinflammation, but rated the evidence as low certainty and acknowledged unresolved randomized evidence [28]. Expert consensus may inform trial design and bedside equipoise, but it does not establish routine efficacy.

The 2026 Surviving Sepsis Campaign suggests against routine blood-purification techniques in adults with sepsis or septic shock because certainty in patient-important benefit remains very low and resource requirements are substantial [29]. This recommendation encompasses heterogeneous modalities, but it appropriately reinforces the need for adequately powered device-specific trials rather than routine adoption on the basis of physiologic signals alone [30].

Recent case reports provide additional clinical context. Sewify et al. described recovery from refractory septic shock during sequential organ support combining oXiris-based CRRT, extracorporeal carbon dioxide removal, and IgM/IgA-enriched immunoglobulin [31]. Alghamdi et al. described rapid norepinephrine de-escalation after oXiris-based CVVHD in a patient with source-controlled septic shock following gallbladder perforation [32]. These observations illustrate the use of oXiris within multimodal critical care and inform hypotheses for further study; concurrent treatments and the absence of comparator groups preclude attribution of recovery to the membrane itself.

### 4.1. Clinical Implications and Future Research

PURE contributes a randomized estimate of 28-day mortality in adults with septic shock and AKI who already require CVVHD. The confidence interval remains wide, while the short-term observations identify hypotheses for further study. They do not, however, provide conclusive evidence for treatment selection or accelerated recovery. Future evaluation should also address inadvertent antibiotic or sedative removal, because routine clinical care and absence of reported device-related events do not substitute for a protocolized drug-exposure assessment.

The decision to withdraw subgroup effect estimates applies to every protocol-listed variable, rather than selectively retaining a favorable norepinephrine result. Baseline vasopressor requirement may be considered as a prespecified stratification variable in a future study, but the present trial does not establish a threshold for treatment selection.

A definitive multicenter trial should use center-stratified randomization, prospectively document protocol and registry amendments, standardize comparator membranes and replacement schedules, and capture prescribed and delivered doses, blood and dialysate flows, net ultrafiltration, actual filter duration, filter counts, and downtime. It should prespecify how death and other intercurrent events enter the analysis of vasopressor-free days and organ recovery, use complete fixed-time follow-up, and adjudicate harms. Protocolized antibiotic and sedative exposure assessment, kidney recovery, health-related quality of life, and cost-effectiveness should be considered.

### 4.2. Strengths and Limitations

Strengths include randomized multicenter recruitment, a clinically defined septic shock–AKI population, registration before first enrollment, complete reported day 28 vital status in the analyzed cohort, and presentation of absolute and relative mortality estimates. Central allocation, concealment, masking procedures, the Prismaflex platform, and the core CVVHD protocol were applied consistently across participating centers. The same oXiris membrane and scheduled replacement regimen were used in the intervention arm at each center. These shared procedures support comparability of intended trial conduct, while the detailed limitations of actual treatment-delivery measurement are reported separately.

Several limitations should be considered. This feasibility-based pilot trial had no formal efficacy sample-size calculation and was not powered to establish a mortality difference. The archived documentation did not specify an interim efficacy analysis or a formal statistical stopping boundary. Recruitment was uneven (162/202 participants at King Saud Medical City and 40/202 at the collaborating ICU), and randomization was not stratified by center. Although the same central allocation and core CVVHD procedures were used at each site, differences in case mix and nonprotocolized clinical care remain possible; no center-adjusted model or separate within-center treatment comparisons are retained in this report. The original registry described a single-center trial, and a dated public-registry update documenting expansion before enrollment ended could not be verified. Clinicians were unblinded, some cointerventions were not protocolized, and baseline prognostic differences were present. Sequence-generation personnel and software were not specified in the available documentation. Two consent withdrawals resulted in a modified rather than strict intention-to-treat analysis; the hypothetical sensitivity scenarios do not replace observed outcomes for those participants.

The absence of centrally captured control-membrane models, surface areas, replacement schedules, delivered dose, achieved blood and dialysate flows, net ultrafiltration, actual filter duration, filter counts, and downtime is a fundamental limitation of internal validity when attributing secondary physiologic differences specifically to the membrane. A common prescription and platform do not establish equivalent delivered treatment. Filter properties, treatment interruptions, and clinician-directed source control, antimicrobials, fluids, corticosteroids, ventilation, and vasopressor titration may also contribute to the observed differences.

The time-specific analysis populations and handling of death, ICU discharge or transfer, vasopressor discontinuation, CRRT cessation, and missed measurements could not be verified for the 72 h outcomes. Death precludes subsequent physiologic measurement and is not equivalent to a zero-vasopressor requirement. Survivor selection, informative missingness, and unequal observation time therefore cannot be assessed. Baseline and 72 h group means may describe different participants; no paired, survivor-specific, or competing-event analysis is reported. Accordingly, secondary outcomes, including length of stay, are presented descriptively without inferential statistics, and formal subgroup inference has not been retained.

The original 10-predictor model was fitted with 87 deaths and did not include center or baseline norepinephrine. This adjusted analysis remains exploratory, and individual covariate coefficients should not be interpreted causally. Multiple-imputation implementation details, including the number of imputations, model specification, and pooling, were not fully documented (Note S1). Archived regression diagnostics are described in Note S2. The Bayesian analysis was post-hoc and used an approximate indirect prior from a trial of a different device and population; its conditional probability does not establish efficacy or overcome limited precision. Harms were investigator-reported without independent adjudication, systematic surveillance for uncommon events, or protocolized antibiotic or sedative concentration monitoring. Absence of reported device-related adverse events therefore does not exclude unrecognized drug underexposure or uncommon harms. Circulating endotoxin and cytokine removal, kidney recovery, outcomes beyond day 28, quality of life, and cost-effectiveness were not assessed.

## 5. Conclusions

In adults with septic shock and AKI requiring CVVHD, this multicenter pilot randomized trial observed numerically lower 28-day mortality with oXiris than with conventional CVVHD, but the difference was not statistically significant. The trial nevertheless provides randomized mortality estimates and practical experience of implementing oXiris-based CVVHD within a shared protocol. Lower reported 72 h norepinephrine requirements, lactate, CRP, and SOFA scores are encouraging signals for further investigation, although these descriptive summaries do not establish accelerated recovery. Together, these findings provide a useful foundation for an adequately powered trial with standardized treatment delivery, prespecified longitudinal methods, complete outcome ascertainment, and systematic safety assessment. Patient-important benefit remains uncertain, and routine adoption is not supported by this pilot trial alone.

## Figures and Tables

**Table 1:** Baseline Characteristics of the Modified Intention-to-Treat Cohort.

Characteristic	Conventional CVVHD (*n* = 102)	oXiris CVVHD (*n* = 100)	Standardized Difference (oXiris − Control)
Age, years	60.2 (5.6)	60.2 (4.6)	0.006
Male sex	65 (63.7)	58 (58.0)	−0.12
Mean arterial pressure, mmHg	59.28 (8.67)	57.32 (9.35)	−0.217
Norepinephrine, µg/kg/min	0.36 (0.10)	0.39 (0.10)	0.30
SOFA score	12.0 (2.5)	11.8 (3.1)	−0.07
PaO_2_/FiO_2_ ratio	229.8 (77.2)	195.5 (73.8)	−0.45
APACHE IV score	100.6 (35.0)	95.6 (40.7)	−0.13
Predicted mortality rate	0.44 (0.20)	0.40 (0.30)	−0.16
Lactate, mmol/L	5.4 (2.5)	5.3 (2.5)	−0.04
White blood cell count, ×10^9^/L	14.5 (4.3)	13.7 (3.2)	−0.21
Platelet count, ×10^9^/L	90.5 (23.6)	85.8 (25.0)	−0.19
Blood urea nitrogen, mg/dL	16.0 (6.3)	15.9 (5.9)	−0.02
Creatinine, µmol/L	172.50 (80.98)	189.84 (73.76)	0.223
C-reactive protein, mg/L	144.22 (11.57)	146.59 (9.98)	0.219
Gram-positive culture	8 (7.8)	7 (7.0)	−0.03
Gram-negative culture	78 (76.5)	76 (76.0)	−0.01

Data are mean (SD) or *n* (%). Standardized differences are calculated as oXiris minus conventional CVVHD: negative values indicate lower values in the oXiris group. They describe the magnitude and direction of imbalance, not statistical significance. Mean arterial pressure, creatinine, and CRP differences round to −0.22, 0.22, and 0.22, respectively; their signs follow this stated convention. Age means round to the same displayed value; its archived standardized difference is retained. APACHE, Acute Physiology and Chronic Health Evaluation; CVVHD, continuous venovenous hemodialysis; SOFA, Sequential Organ Failure Assessment.

**Table 2:** Primary Mortality Outcome and Descriptive Secondary Summaries.

Outcome	Conventional CVVHD	oXiris CVVHD	Primary Effect Estimate/Reporting Scope
28-day mortality	48/102 (47.1)	39/100 (39.0)	RR 0.83 (0.60–1.14); RD −8.1 percentage points (−21.7 to 5.6); *p* = 0.247
ICU length of stay, days	16.1 (7.9)	13.7 (6.5)	
Hospital length of stay, days	21.4 (11.2)	20.1 (9.9)	
Norepinephrine at 72 h, µg/kg/min	0.25 (0.20)	0.18 (0.20)	
Lactate at 72 h, mmol/L	2.8 (1.7)	2.2 (1.5)	
SOFA score at 72 h	3.5 (1.0)	3.1 (1.0)	
C-reactive protein at 72 h, mg/L	75.2 (40.8)	54.6 (29.0)	
Creatinine at 72 h, µmol/L	115.4 (6.6)	114.7 (6.3)	
Blood urea nitrogen at 72 h, mg/dL	6.8 (1.7)	6.5 (2.3)	
White blood cell count at 72 h, ×10^9^/L	10.1 (2.1)	10.4 (2.0)	

The denominators in the mortality row refer to the modified intention-to-treat cohort with reported day 28 vital status. They are not the numbers assessed for secondary outcomes. Secondary values reproduce the reported means and standard deviations; their time-specific analysis populations and rules for death, vasopressor discontinuation, ICU discharge or transfer, CRRT cessation, and missed measurements could not be verified. No secondary confidence intervals, *p*-values, or model-based treatment effects are presented. See Section 4.2 and Note S4. Data are *n*/total (%) for mortality and reported mean (SD) for secondary summaries. The primary risk ratio and risk difference compare oXiris with conventional CVVHD. CI, confidence interval; ICU, intensive care unit; RD, risk difference; RR, risk ratio; SD, standard deviation; SOFA, Sequential Organ Failure Assessment.

**Table 3:** Covariate-Adjusted Sensitivity Analysis for 28-Day Mortality.

Variable	Adjusted OR	95% CI	*p*-Value
Group (Intervention vs. Control)	0.610	0.326–1.141	0.122
Age (per year)	1.067	1.005–1.133	0.034 *
Mean Arterial Pressure (per mmHg)	1.031	0.996–1.066	0.080
PaO_2_/FiO_2_ (per unit)	0.995	0.991–0.999	0.015 *
Lactate (per mmol/L)	0.989	0.877–1.115	0.855
Creatinine (per µmol/L)	1.001	0.997–1.005	0.520
Platelets (per ×10^9^/L)	1.010	0.997–1.022	0.124
C-Reactive Protein (per mg/L)	0.996	0.969–1.024	0.800
White Blood Cells (per ×10^9^/L)	0.945	0.871–1.025	0.172
APACHE IV (per point)	0.999	0.992–1.007	0.881

OR, odds ratio; CI, confidence interval. The original 10-covariate model included 87 deaths and is exploratory; individual covariate coefficients should not be interpreted causally. * *p* < 0.05 (nominal).

## Data Availability

The supplementary archive contains the reported aggregate summaries, regression-output summaries, and code for the aggregate calculations. The corresponding author is the contact for requests concerning retained participant-level records, the data dictionary, protocol, and original statistical files, subject to institutional approval, privacy requirements, and a data-use agreement. Availability of a complete linked longitudinal dataset is not established by these materials. Note S1 distinguishes the documentation available for this report from the information needed to reproduce participant-level analyses; aggregate summaries are not a substitute for the original analysis file or log.

## Supplementary Materials

The following supporting information can be downloaded at: https://www.mdpi.com/article/10.3390/jcm15208028/s1. Notes S1–S6 cover trial and analysis documentation; archived regression and diagnostics; the post hoc unadjusted Bayesian analysis; descriptive secondary outcomes and subgroup reporting; hypothetical consent-withdrawal sensitivity scenarios; and side-by-side baseline and 72-h summaries. Tables S1–S14 provide the corresponding summaries and original model outputs. Figures S1–S3 show the archived adjusted-model forest plot, archived ROC curve, and Bayesian prior sensitivity. Code S1 and the aggregate-results JSON file reproduce the binary-outcome, Bayesian, and withdrawal-scenario calculations. Code S2 and Data S1 verify the descriptive source means displayed in Table S14; they do not calculate individual changes or inferential statistics. The file index identifies every supplementary item.

## Abbreviations

AKI	acute kidney injury
APACHE	Acute Physiology and Chronic Health Evaluation
CI	confidence interval
CRP	C-reactive protein
CRRT	continuous renal replacement therapy
CVVHD	continuous venovenous hemodialysis
ICU	intensive care unit
MAP	mean arterial pressure
PaO_2_/FiO_2_	ratio of arterial oxygen partial pressure to inspired oxygen fraction
RD	risk difference
RR	risk ratio
SOFA	Sequential Organ Failure Assessment
ARDS	acute respiratory distress syndrome
AUC	area under the curve
OR	odds ratio
ROC	receiver operating characteristic
SD	standard deviation
