# Supplementary material for: oXiris-Based Blood Purification Efficacy in Adults with Septic Shock and Acute Kidney Injury: The PURE Multicenter Pilot Randomized Clinical Trial
Source: J Clin Med. 2026 Oct 16;15(20):8028. doi: 10.3390/jcm15208028 (PMC13706304; doi:10.3390/jcm15208028)
Supplement: Supplementary file 1 [file jcm-15-08028-s001.zip › 00_Supplementary_File_Index.pdf]

Supplementary file index

28 separate files: 6 notes, 14 editable tables, 3 PNG figures, 2 Python scripts, 1 JSON results file, 1 CSV file, and this index. The original adjusted sensitivity analysis remains distinct from the primary unadjusted randomized comparison. Secondary outcome summaries are descriptive only.

Notes S1–S6

Item	Content	Exact filename
Note S1	Trial documentation and reported missing-data procedures	Note_S1_Trial_Documentation.pdf
Note S2	Original adjusted regression and diagnostic limitations	Note_S2_Archived_Regression.pdf
Note S3	Post hoc unadjusted Bayesian methods	Note_S3_Bayesian_Methods.pdf
Note S4	Descriptive secondary outcomes and corrected subgroup reporting	Note_S4_Secondary_Outcome_Limitations.pdf
Note S5	Hypothetical consent-withdrawal sensitivity scenarios	Note_S5_Withdrawal_Sensitivity.pdf
Note S6	Side-by-side baseline and 72-hour summaries	Note_S6_Baseline_72h_Comparison.pdf

Tables S1–S14

Item	Content	Exact filename
Table S1	CVVHD delivery and safety	Table_S1_CVVHD_Delivery_and_Safety.pdf
Table S2	Sources of infection	Table_S2_Infection_Sources.pdf
Table S3	Original univariable logistic regression	Table_S3_Archived_univariable_logistic_regression.pdf
Table S4	Original variance inflation factors	Table_S4_Archived_variance_inflation_factors.pdf
Table S5	Original full covariate-adjusted model	Table_S5_Archived_full_covariate-adjusted_model.pdf
Table S6	Original classification table	Table_S6_Archived_classification_table.pdf
Table S7	Original Box–Tidwell output	Table_S7_Archived_Box–Tidwell_output.pdf
Table S8	Unadjusted Bayesian reference analysis	Table_S8_Unadjusted_Bayesian_Results.pdf
Table S9	Bayesian prior sensitivity	Table_S9_Bayesian_Prior_Sensitivity.pdf
Table S10	Descriptive norepinephrine AUC	Table_S10_Archived_Norepinephrine_AUC.pdf
Table S11	Reported norepinephrine means at nominal time points	Table_S11_Archived_Norepinephrine_Timepoints.pdf
Table S12	Subgroup variables and reporting status	Table_S12_Subgroup_Reporting.pdf
Table S13	Hypothetical consent-withdrawal bounds	Table_S13_Hypothetical_Withdrawal_Bounds.pdf
Table S14	Side-by-side baseline and 72-hour means	Table_S14_Baseline_and_72h_Descriptive_Summaries.pdf

Figures, code, and data files

Item	Content	Exact filename
Figure S1	Original adjusted-model forest plot	Figure_S1.png
Figure S2	Original apparent ROC curve	Figure_S2.png
Figure S3	Post hoc Bayesian prior sensitivity	Figure_S3.png
Code S1	Aggregate primary, Bayesian and withdrawal checks	Code_S1_Aggregate_Reproducibility.py
Results JSON	Reference numerical results for Code S1	Aggregate_Check_Results.json
Code S2	Consistency check of the descriptive Table S14 means	Code_S2_Verify_Descriptive_Source_Means.py
Table S14 CSV	Reported source means; no change-score columns	Table_S14_Reported_Group_Means.csv

Supplementary figure legends

Figure S1. Original adjusted odds ratios and 95% confidence intervals for 28-day mortality. These exploratory estimates are subject to the diagnostic and imputation limitations in Note S2.

Figure S2. Original apparent receiver-operating-characteristic curve for the adjusted logistic model (AUC = 0.681). This is an in-sample diagnostic, not external validation.

Figure S3. Posterior probability that the unadjusted mortality odds ratio is below 1 across retained precision of the indirect Tigris prior. The analysis is post hoc and contains no confirmatory success threshold.

Running the reproducibility scripts

Extract the archive before running the scripts; keep each script with its input file. Both scripts use Python standard-library modules.

```
python Code_S1_Aggregate_Reproducibility.py
python Code_S2_Verify_Descriptive_Source_Means.py
```

Code S1 reproduces the aggregate numerical calculations associated with Aggregate_Check_Results.json. Code S2 checks the Table S14 source means only. Neither script reproduces a participant-level regression or verifies a source-record mortality audit.
