# Supplementary material for: oXiris-Based Blood Purification Efficacy in Adults with Septic Shock and Acute Kidney Injury: The PURE Multicenter Pilot Randomized Clinical Trial
Source: J Clin Med. 2026 Oct 16;15(20):8028. doi: 10.3390/jcm15208028 (PMC13706304; doi:10.3390/jcm15208028)
Supplement: Supplementary file 1 [file jcm-15-08028-s001.zip › Note_S1_Trial_Documentation.pdf]

Supplementary Note S1. Trial documentation and missing data

PURE was registered with Research Registry on 22 December 2024 (researchregistry10932), before first enrollment on 21 January 2025. The original entry described a single-center trial; the completed study recruited at King Saud Medical City and a collaborating ICU. The randomized comparison and primary endpoint of 28-day all-cause mortality remained unchanged. A dated registry update documenting the additional center before enrollment ended was not available for verification. The public registry history is distinct from institutional continuing-review approval.

The King Saud Medical City IRB reference is H1RI-24-Jul22-01. The approval memorandum is dated 04-09-2022. Continuing-approval notices record validity from 16-01-2025 to 15-07-2025 and from 16-07-2025 to 15-01-2026. Together, the latter periods encompass recruitment from 21-01-2025 through 13-11-2025. The original notices are supplied separately for confidential editorial review rather than as public supplementary material.

All participating centers followed the same central telephone 1:1 randomization procedure in blocks of four, with allocation concealed until eligibility confirmation. Treating clinicians were unmasked because circuit preparation differed between groups; participants or their surrogates were masked, and the statistical analyst received coded group labels. Both treatment groups used the Prismaflex platform and the same core CVVHD protocol at each center: a prescribed effluent dose of 20–30 mL/kg/h, circuit unfractionated heparin, and double-lumen 12 Fr jugular or femoral access. The intervention arm used the same oXiris membrane, blood-flow range of 100–200 mL/min, and replacement every 24 h for up to 72 h while treatment continued. Control membrane selection and replacement followed local practice. These cross-center procedures were confirmed by the investigators. Actual delivered dose, downtime, and detailed control-filter measurements were not captured centrally; the shared protocol and platform do not establish equivalence of those unmeasured exposures.

Individual 1:1 randomization used central telephone allocation in blocks of four, with concealment until eligibility confirmation. The same unstratified scheme continued after recruitment expanded; center-specific allocation sequences were not introduced. Of 202 analyzed participants, 162 (80.2%) were recruited at King Saud Medical City and 40 (19.8%) at the collaborating ICU. The original adjusted sensitivity model does not include center. No additional center-adjusted model or separate within-center treatment comparisons are retained in the final report.

The reported screening flow is 3334 ICU admissions, 697 patients with CRRT ordered, 251 potentially eligible patients with AKI and septic shock, and 204 randomized participants. Two participants allocated to oXiris withdrew permission for continued data use, leaving 100 oXiris and 102 conventional-CVVHD participants. Day-28 vital status was reported for all 202 analyzed participants. No other mortality time point is reported as an endpoint in this article.

Isolated laboratory measurements were missing in three control participants and two oXiris participants. The source reports multiple imputation under the planned rule for less than 10% missingness. The number of imputations, variables in the imputation model, and pooling details were not retained. No replacement participant-level imputation has been performed for this revision.

Notes S2–S5 distinguish the reported original analyses from the aggregate calculations supplied for reproducibility. Their availability does not substitute for the participant-level dataset, protocol history, or original statistical output.
