# Supplementary material for: oXiris-Based Blood Purification Efficacy in Adults with Septic Shock and Acute Kidney Injury: The PURE Multicenter Pilot Randomized Clinical Trial
Source: J Clin Med. 2026 Oct 16;15(20):8028. doi: 10.3390/jcm15208028 (PMC13706304; doi:10.3390/jcm15208028)
Supplement: Supplementary file 1 [file jcm-15-08028-s001.zip › Note_S2_Archived_Regression.pdf]

Supplementary Note S2. Archived logistic model and diagnostic limitations

Tables S3–S7 and Figures S1–S2 present the original adjusted analysis and its diagnostic summaries. This original analysis is exploratory and supplements, rather than replaces, the unadjusted randomized mortality comparison.

The full model included treatment, age, mean arterial pressure, PaO_2/FiO_2, lactate, creatinine, platelets, C-reactive protein, white blood cell count, and APACHE IV score. It reported an adjusted treatment odds ratio of 0.610 (95% CI, 0.326–1.141; $p = 0.122$) with 87 deaths. Center and baseline norepinephrine were not included. Low variance inflation factors do not resolve possible overfitting, residual imbalance, or uncertainty about the missing-data procedure.

The source reported likelihood-ratio $\chi^2 = 20.96$ (10 degrees of freedom; $p = 0.021$) and pseudo-$R^2 = 0.076$. Its narrative simultaneously called the latter “Nagelkerke-style” and “McFadden”; because those measures are not interchangeable and the original software output is unavailable, the precise pseudo-R^2 definition is not asserted here.

The Hosmer–Lemeshow result was $\chi^2 = 5.85$ (8 degrees of freedom; $p = 0.664$). Failure to reject this test does not establish calibration. The archived ROC area was 0.681; the source did not supply a numerical confidence interval or an external validation set. The model should not be described as having established clinical predictive performance.

The source diagnostic narrative explicitly reports that creatinine was excluded from Box–Tidwell testing because of an implausible negative value. The affected source record, whether this reflected entry, transformation, or imputation, and its handling in the adjusted model are not available for verification. No correction or successful data-validation exercise is claimed. This limits reliance on the adjusted model; the reported unadjusted mortality comparison does not use creatinine.

The archived maximum Cook’s distance was 0.033, with six observations reportedly exceeding $4/202 \approx 0.0198$. The original statement that 0.033 was below 0.0198 was incorrect; the numbers are retained without that assertion. Neither a small maximum Cook’s distance nor a nonsignificant Box–Tidwell term proves that the model is valid. No participant-level sensitivity refit excluding flagged observations was available.

Figure S1. Archived covariate-adjusted odds ratios and 95% confidence intervals for 28-day mortality. The figure is retained as supplied output, subject to the diagnostic and imputation limitations above.

Figure S2. Archived receiver-operating-characteristic curve for the full logistic model (AUC = 0.681). This is an apparent, in-sample diagnostic, not external validation.
