# Supplementary material for: oXiris-Based Blood Purification Efficacy in Adults with Septic Shock and Acute Kidney Injury: The PURE Multicenter Pilot Randomized Clinical Trial
Source: J Clin Med. 2026 Oct 16;15(20):8028. doi: 10.3390/jcm15208028 (PMC13706304; doi:10.3390/jcm15208028)
Supplement: Supplementary file 1 [file jcm-15-08028-s001.zip › Note_S3_Bayesian_Methods.pdf]

Supplementary Note S3. Post hoc unadjusted Bayesian sensitivity analysis

The retained calculation is a normal–normal approximation on log odds ratios, using only the reported PURE mortality counts: 39 deaths and 61 survivors with oXiris; 48 deaths and 54 survivors with conventional CVVHD. These give an unadjusted odds ratio of 0.719262, log odds ratio −0.329529, and standard error 0.285283.

The indirect source is the Tigris-only 28-day adjusted odds ratio of 0.80 (95% credible interval, 0.38–1.49). Tigris evaluated a different device and population. Its interval is a credible interval, not a frequentist confidence interval. We approximate its log-scale standard deviation by $[\ln(1.49) - \ln(0.38)]/(2 \times 1.959964)$ and center the approximation at $\ln(0.80)$. This width-matched approximation is not an exact reproduction of the Tigris posterior or its individual-level model.

For retained precision w, prior precision is w/s_0^2 and likelihood precision is $1/s^2$. The posterior variance is $V = 1/(1/s^2 + w/s_0^2)$, and posterior mean is $M = V[y/s^2 + w\mu_0/s_0^2]$. The posterior odds ratio is exp(M), with a 95% credible interval $\exp[M \pm 1.959964\sqrt{V}]$. The reported probability is $P(OR < 1) = \Phi(-M/\sqrt{V})$, conditional on this approximate model.

The 75% reference scenario gives posterior OR 0.745 (95% CrI, 0.472–1.176) and P(OR < 1) = 89.7%. Across w = 0, 0.25, 0.50, 0.75, and 1, the probability ranges from 87.6% to 90.3%. At w = 0, the calculation is the data-only normal likelihood with a flat prior on the log-odds scale. The 75% weight is a post hoc reference choice; no prespecified 95% success criterion is applied.

The earlier supplement labeled a likelihood OR of 0.713 (95% CI, 0.407–1.249) as “adjusted” and combined it with the prior to obtain OR 0.741 and 90.1% probability of benefit. That likelihood could not be mapped to the separately reported full adjusted model OR 0.610 (95% CI, 0.326–1.141). The adjusted Bayesian row, its weight-sensitivity results, and the corresponding old graphic are therefore not retained. This is an identified discrepancy, not an assumption that either adjusted likelihood is correct. No new adjusted Bayesian model was fitted.

Code S1 provides a dependency-free reproducibility script for the unadjusted binary estimates, this Bayesian approximation, and the hypothetical withdrawal bounds. It does not create individual trial records. Table S8, Table S9, and Figure S3 report its aggregate output.

Source: Neyra JA, Legrand M, Tidswell MA, et al. Polymyxin B haemoadsorption in endotoxic septic shock (Tigris): a multicentre, open-label, Bayesian, randomised, controlled, phase 3 trial. Lancet Respir Med. 2026;14(5):443–452. doi:10.1016/S2213-2600(26)00047-0.

Figure S3. Posterior probability that the mortality odds ratio is below 1 across retained precision of the indirect Tigris prior. The analysis is post hoc; the figure contains no confirmatory success threshold.
