# Supplementary material for: oXiris-Based Blood Purification Efficacy in Adults with Septic Shock and Acute Kidney Injury: The PURE Multicenter Pilot Randomized Clinical Trial
Source: J Clin Med. 2026 Oct 16;15(20):8028. doi: 10.3390/jcm15208028 (PMC13706304; doi:10.3390/jcm15208028)
Supplement: Supplementary file 1 [file jcm-15-08028-s001.zip › Note_S4_Secondary_Outcome_Limitations.pdf]

Supplementary Note S4. Secondary outcomes and subgroup reporting

The earlier norepinephrine summary reported 2626 observations: 202 participants multiplied by 13 nominal time points from 0 to 72 h. This arithmetic does not establish that every participant was alive, receiving treatment, or assessed at every time. Time-specific denominators and coding after death, vasopressor discontinuation, ICU discharge or transfer, CRRT cessation, or missed measurement were not preserved in the available summaries.

No zero-after-death, last-observation-carried-forward, or survivor-only rule has been assigned retrospectively. The available summaries do not support reconstruction of survivor-specific, composite, joint, or competing-event analyses. Secondary physiologic and length-of-stay outcomes are reported descriptively, without inferential confidence intervals or p-values. Tables S10–S11 and main Figure 3 retain descriptive summaries only; uncertainty bars and standard errors are not presented. Table S14 displays baseline and 72-hour means side by side, without arithmetic-change columns or paired-change inference.

Following peer review, formal subgroup effect estimates, subgroup interaction tests, AUC significance tests, and inferential mixed-effects results have been removed from the revised report. This is a change in reporting, not a claim that the earlier analyses were never performed. It applies to all subgroup variables and reflects limited power, multiplicity, and incomplete characterization of intercurrent events.

The investigator has clarified the earlier age-stratum reporting error and supplied corrected counts that agree with the reported primary mortality totals. The later statement that a retrospective correction was not possible is superseded by this clarification. The primary mortality totals are unchanged. Age remains in the baseline table, the protocol-listed subgroup variables, and the original adjusted model. No age-specific treatment-effect estimates or subgroup interactions are reinstated. The detailed count reconciliation is provided in the editorial response; Table S12 summarizes the reporting decision.
