# Supplementary material for: oXiris-Based Blood Purification Efficacy in Adults with Septic Shock and Acute Kidney Injury: The PURE Multicenter Pilot Randomized Clinical Trial
Source: J Clin Med. 2026 Oct 16;15(20):8028. doi: 10.3390/jcm15208028 (PMC13706304; doi:10.3390/jcm15208028)
Supplement: Supplementary file 1 [file jcm-15-08028-s001.zip › Note_S5_Withdrawal_Sensitivity.pdf]

Supplementary Note S5. Hypothetical aggregate bounds for consent withdrawals

Two participants allocated to oXiris withdrew permission for continued data use. A true all-randomized participant-level intention-to-treat analysis was not available and was not reconstructed. Their actual day-28 outcomes are not inferred or reported.

To show the numerical influence of two unavailable outcomes, a post hoc aggregate calculation considers three hypothetical scenarios: both survive, one dies, or both die. The oXiris denominator is 102 in these scenarios, with 39, 40, or 41 assumed deaths; the control group remains 48/102. These are mathematical bounds derived from published counts, not a recovery of withdrawn records, observed ITT results, or additional patient data.

Risk differences use independent-binomial Wald intervals; risk-ratio intervals use the log-Wald method. Code S1 specifies the formulas and returns each scenario. The risk difference ranges from −8.8 to −6.9 percentage points; each 95% interval includes no effect. These scenarios do not eliminate bias from post-randomization exclusion and do not remedy other internal-validity limitations.
