# Supplementary material for: oXiris-Based Blood Purification Efficacy in Adults with Septic Shock and Acute Kidney Injury: The PURE Multicenter Pilot Randomized Clinical Trial
Source: J Clin Med. 2026 Oct 16;15(20):8028. doi: 10.3390/jcm15208028 (PMC13706304; doi:10.3390/jcm15208028)
Supplement: Supplementary file 1 [file jcm-15-08028-s001.zip › Note_S6_Baseline_72h_Comparison.pdf]

Supplementary Note S6. Baseline and 72-hour descriptive summaries

Purpose

Table S14 displays reported trial-baseline and 72-hour group means side by side. This retains the descriptive information while avoiding interpretation of marginal subtraction as an analysis of individual change.

Time origin

The baseline summaries concern trial baseline around enrollment and CRRT initiation. They are not relabeled as a separate ICU-admission assessment. Mean arterial pressure and PaO_2/FiO_2 have baseline summaries but no corresponding 72-hour means in the submitted outcome table.

Analysis population

The time-specific denominators and the rules for death, vasopressor discontinuation, ICU discharge or transfer, CRRT cessation, and missing measurements could not be verified. Baseline and 72-hour means may therefore describe different participants. Neither complete follow-up nor a survivor-only cohort is assumed.

Reporting scope

No arithmetic-change columns, paired-change estimates, confidence intervals, p-values, or baseline-adjusted participant-level treatment effects are presented. The descriptive observations do not establish faster recovery or a treatment effect.

Reproducibility

Code S2 checks the CSV values against the reported source means reproduced in Table S14. It does not generate observations, assign analysis denominators, infer intercurrent-event handling, or calculate individual changes or inferential statistics.

Abbreviations: CRRT, continuous renal replacement therapy; ICU, intensive care unit; PaO_2, arterial oxygen partial pressure; FiO_2, inspired oxygen fraction; CSV, comma-separated values.
