# Supplementary material for: oXiris-Based Blood Purification Efficacy in Adults with Septic Shock and Acute Kidney Injury: The PURE Multicenter Pilot Randomized Clinical Trial
Source: J Clin Med. 2026 Oct 16;15(20):8028. doi: 10.3390/jcm15208028 (PMC13706304; doi:10.3390/jcm15208028)
Supplement: Supplementary file 1 [file jcm-15-08028-s001.zip › Table_S10_Archived_Norepinephrine_AUC.pdf]

Table S10. Descriptive reported norepinephrine AUC

The values below reproduce the reported area-under-the-curve summaries. A complete-follow-up analysis denominator and the intercurrent-event rules could not be verified. No AUC hypothesis test, confidence interval, or treatment-effect estimate is presented.

Summary	Conventional CVVHD	oXiris CVVHD
Reported mean AUC	25.5	21.3
Reported SD	4.1	7.7

AUC was described as trapezoidal integration of norepinephrine dose (µg/kg/min) against time (hours). These summaries do not establish faster vasopressor weaning.

Abbreviations: AUC, area under the dose–time curve; SD, standard deviation; CVVHD, continuous venovenous hemodialysis.
