# Supplementary material for: oXiris-Based Blood Purification Efficacy in Adults with Septic Shock and Acute Kidney Injury: The PURE Multicenter Pilot Randomized Clinical Trial
Source: J Clin Med. 2026 Oct 16;15(20):8028. doi: 10.3390/jcm15208028 (PMC13706304; doi:10.3390/jcm15208028)
Supplement: Supplementary file 1 [file jcm-15-08028-s001.zip › Table_S11_Archived_Norepinephrine_Timepoints.pdf]

Table S11. Reported mean norepinephrine dose by nominal time point

Dose unit: µg/kg/min. The means are descriptive summaries. Time-specific denominators and the handling of death, vasopressor discontinuation, ICU discharge or transfer, CRRT cessation, and missed measurement could not be verified. Contributing participants may differ between time points; no standard errors or uncertainty intervals are displayed.

Time, h	Conventional CVVHD mean	oXiris CVVHD mean
0	0.364	0.392
6	0.454	0.471
12	0.445	0.415
18	0.420	0.373
24	0.356	0.301
30	0.342	0.282
36	0.347	0.254
42	0.357	0.245
48	0.340	0.254
54	0.344	0.226
60	0.289	0.223
66	0.248	0.212
72	0.252	0.182

Abbreviations: CVVHD, continuous venovenous hemodialysis; h, hours; ICU, intensive care unit; CRRT, continuous renal replacement therapy.
