# Supplementary material for: oXiris-Based Blood Purification Efficacy in Adults with Septic Shock and Acute Kidney Injury: The PURE Multicenter Pilot Randomized Clinical Trial
Source: J Clin Med. 2026 Oct 16;15(20):8028. doi: 10.3390/jcm15208028 (PMC13706304; doi:10.3390/jcm15208028)
Supplement: Supplementary file 1 [file jcm-15-08028-s001.zip › Table_S12_Subgroup_Reporting.pdf]

Table S12. Subgroup variables and reporting decisions

Protocol-listed variables are retained in the study description. The revised report does not present subgroup risk estimates, interaction p-values, or treatment-selection claims for any stratum. This approach applies consistently to age and the other subgroup variables.

Variable	Protocol-listed grouping	Retained role in the revised report
Sex	Male / female	Baseline description; no treatment-effect interaction reported
Age	At or below / above cohort median	Baseline description and continuous predictor in the adjusted model; no subgroup effect estimate reported
APACHE IV score	At or below / above cohort median	Baseline description and predictor in the adjusted model; no subgroup effect estimate reported
Baseline norepinephrine	At or below / above cohort median	Baseline description; no treatment-responsive threshold or interaction reported
ARDS severity	None/mild / moderate/severe	Protocol-listed subgroup variable; no subgroup effect estimate reported

The investigator supplied corrected age-stratum counts that agree with the reported primary mortality totals. Earlier wording that described the count discrepancy as unresolved has been superseded. The detailed reconciliation is provided to the editor; this table reports subgroup-analysis status, not new subgroup treatment effects. See Note S4.

Abbreviations: APACHE IV, Acute Physiology and Chronic Health Evaluation IV; ARDS, acute respiratory distress syndrome.
