# Supplementary material for: oXiris-Based Blood Purification Efficacy in Adults with Septic Shock and Acute Kidney Injury: The PURE Multicenter Pilot Randomized Clinical Trial
Source: J Clin Med. 2026 Oct 16;15(20):8028. doi: 10.3390/jcm15208028 (PMC13706304; doi:10.3390/jcm15208028)
Supplement: Supplementary file 1 [file jcm-15-08028-s001.zip › Table_S13_Hypothetical_Withdrawal_Bounds.pdf]

Table S13. Hypothetical aggregate sensitivity to two consent withdrawals

Hypothetical status of withdrawals	Assumed oXiris deaths / n	RR (95% CI)	RD, percentage points (95% CI)
Neither died	39/102	0.81 (0.59–1.12)	-8.8 (-22.3 to 4.7)
One died	40/102	0.83 (0.61–1.14)	-7.8 (-21.4 to 5.7)
Both died	41/102	0.85 (0.62–1.17)	-6.9 (-20.4 to 6.7)

These are newly calculated hypothetical scenarios, not observed outcomes and not a true participant-level ITT analysis. The control group remains 48/102 in all scenarios. No withdrawn records were used. Confidence intervals follow Code S1; all include no effect.

Abbreviations: ITT, intention-to-treat; n, number of participants; RR, risk ratio; RD, risk difference; CI, confidence interval.
