# Supplementary material for: oXiris-Based Blood Purification Efficacy in Adults with Septic Shock and Acute Kidney Injury: The PURE Multicenter Pilot Randomized Clinical Trial
Source: J Clin Med. 2026 Oct 16;15(20):8028. doi: 10.3390/jcm15208028 (PMC13706304; doi:10.3390/jcm15208028)
Supplement: Supplementary file 1 [file jcm-15-08028-s001.zip › Table_S14_Baseline_and_72h_Descriptive_Summaries.pdf]

Table S14. Reported baseline and 72-hour group means

This table displays the reported means from manuscript Tables 1 and 2 side by side. It does not estimate individual change, compare paired recovery, or provide a baseline-adjusted treatment effect.

Parameter (unit)	Conventional CVVHD baseline	Conventional CVVHD 72 h	oXiris CVVHD baseline	oXiris CVVHD 72 h
Norepinephrine (µg/kg/min)	0.36	0.25	0.39	0.18
Lactate (mmol/L)	5.4	2.8	5.3	2.2
SOFA score (points)	12.0	3.5	11.8	3.1
C-reactive protein (mg/L)	144.22	75.2	146.59	54.6
Creatinine (µmol/L)	172.50	115.4	189.84	114.7
Blood urea nitrogen (mg/dL)	16.0	6.8	15.9	6.5
White blood cell count ($\times 10^9$/L)	14.5	10.1	13.7	10.4
Mean arterial pressure (mmHg)	59.28	Not reported	57.32	Not reported
PaO_2/FiO_2 ratio (ratio)	229.8	Not reported	195.5	Not reported

Baseline denotes trial baseline around enrollment/CRRT initiation, not a separately verified ICU-admission measurement. Baseline and 72-hour summaries may describe different participants. The full-cohort totals must not be used as paired or time-specific sample sizes. No arithmetic-change columns, confidence intervals, p-values, imputed correlations, or post-death substitutions are presented. See Note S6.

Abbreviations: CVVHD, continuous venovenous hemodialysis; CRRT, continuous renal replacement therapy; ICU, intensive care unit; SOFA, Sequential Organ Failure Assessment; PaO_2, arterial oxygen partial pressure; FiO_2, inspired oxygen fraction; h, hours.
