# Supplementary material for: oXiris-Based Blood Purification Efficacy in Adults with Septic Shock and Acute Kidney Injury: The PURE Multicenter Pilot Randomized Clinical Trial
Source: J Clin Med. 2026 Oct 16;15(20):8028. doi: 10.3390/jcm15208028 (PMC13706304; doi:10.3390/jcm15208028)
Supplement: Supplementary file 1 [file jcm-15-08028-s001.zip › Table_S1_CVVHD_Delivery_and_Safety.pdf]

Table S1. CVVHD delivery, data availability, and safety

Characteristic	Conventional CVVHD	oXiris CVVHD	Evidence / limitation
Modality	CVVHD	CVVHD	CVVHD used in both arms across participating centers
Platform	Prismaflex	Prismaflex	Same Prismaflex platform in both arms at every participating center; group-specific machine logs not retained centrally
Exact membrane model	Not captured centrally	oXiris	Same oXiris membrane across centers; no control model inferred from compatibility
Membrane surface area	Not available	Not captured as trial data	Commercial specifications are not substituted for trial records
Prescribed effluent dose	20–30 mL/kg/h	20–30 mL/kg/h	Shared target across centers; actual delivered dose not captured centrally
Vascular access	Double-lumen 12 Fr catheter; internal jugular or femoral vein	Double-lumen 12 Fr catheter; internal jugular or femoral vein	Shared access criteria across centers
Delivered effluent dose	Not captured centrally	Not captured centrally	No group-specific exposure comparison
Blood flow	Not available as group-specific data	Specified range 100–200 mL/min	Actual time-weighted delivery not available
Dialysate flow	Not captured centrally	Not captured centrally	Not inferred from prescribed weight-normalized dose
Net ultrafiltration	Not captured centrally	Not captured centrally	Fluid removal and balance not reconstructable
Replacement schedule	Local practice; exact schedule unavailable	Every 24 h for up to 72 h while treatment continued	The stated oXiris schedule applied at each center; detailed delivered exposure unavailable
Actual filter duration	Not captured centrally	Not captured centrally	No filter survival analysis possible
Number of filters	Not captured centrally	Not captured centrally	Not inferred as three filters per participant
Circuit downtime	Not captured centrally	Not captured centrally	No uptime-adjusted delivered-dose analysis
Anticoagulation	Unfractionated heparin	Unfractionated heparin	Circuit administration across centers; cumulative exposure not available
Premature filter clotting within 72 h	7/102 (6.9%)	8/100 (8.0%)	Investigator-reported participant counts, not circuit-level rates
Device-related adverse events	None reported	None reported	No independent adjudication; absence of reported events is not established safety

Characteristic	Conventional CVVHD	oXiris CVVHD	Evidence / limitation
Antibiotic / sedative adsorption	Systematic monitoring not documented	Systematic monitoring not documented	No trial-specific drug-level or extraction dataset available
Filter supply	Routine hospital inventory	Manufacturer in-kind supply	Manufacturer reported to have no role in trial conduct, analysis, or reporting

All participating centers used the same central allocation and masking procedures, the Prismaflex platform in both treatment groups, and the same core CVVHD protocol. The intervention arm used the same oXiris membrane and scheduled replacement regimen at each center. This table distinguishes those investigator-confirmed procedures from actual treatment-delivery measurements that were not captured centrally. Missing process data limit attribution of secondary physiologic differences specifically to the membrane.

Abbreviations: CVVHD, continuous venovenous hemodialysis; CRRT, continuous renal replacement therapy; Fr, French catheter-size unit; h, hour(s).
