# Supplementary material for: oXiris-Based Blood Purification Efficacy in Adults with Septic Shock and Acute Kidney Injury: The PURE Multicenter Pilot Randomized Clinical Trial
Source: J Clin Med. 2026 Oct 16;15(20):8028. doi: 10.3390/jcm15208028 (PMC13706304; doi:10.3390/jcm15208028)
Supplement: Supplementary file 1 [file jcm-15-08028-s001.zip › Table_S2_Infection_Sources.pdf]

Table S2. Sources of infection

Source	Conventional CVVHD (n = 102)	oXiris CVVHD (n = 100)
Positive culture	93 (91.2)	89 (89.0)
Pulmonary	35 (34.3)	28 (28.0)
Abdominal	52 (51.0)	58 (58.0)
Blood	8 (7.8)	7 (7.0)
Skin/soft tissue	5 (4.9)	6 (6.0)
Urine	2 (2.0)	1 (1.0)

Data are n (%). Some participants had more than one positive culture; source categories may overlap. Source values are retained without new between-group tests.

Abbreviations: CVVHD, continuous venovenous hemodialysis; n, number of participants.
