# Supplementary material for: oXiris-Based Blood Purification Efficacy in Adults with Septic Shock and Acute Kidney Injury: The PURE Multicenter Pilot Randomized Clinical Trial
Source: J Clin Med. 2026 Oct 16;15(20):8028. doi: 10.3390/jcm15208028 (PMC13706304; doi:10.3390/jcm15208028)
Supplement: Supplementary file 1 [file jcm-15-08028-s001.zip › Table_S3_Archived_univariable_logistic_regression.pdf]

Table S3. Archived univariable logistic regression

Variable	Unadj. OR	95% CI	p-value
Group (1 vs 0)	0.719	0.411 – 1.258	0.248
Age	1.070	1.011 – 1.132	0.020
MAP	1.029	0.997 – 1.062	0.078
P/F ratio	0.996	0.992 – 1.000	0.036
Lactate	1.032	0.924 – 1.153	0.576
Creatinine	1.001	0.997 – 1.005	0.606
Platelets	1.009	0.997 – 1.021	0.129
CRP	0.995	0.970 – 1.021	0.713
WBC	0.948	0.880 – 1.021	0.155
APACHE IV	0.999	0.991 – 1.006	0.712

Odds ratios and p-values are retained from the source output, without refitting. These are exploratory associations; no causal covariate interpretation is intended.

Abbreviations: Unadj., unadjusted; OR, odds ratio; CI, confidence interval; MAP, mean arterial pressure; P/F ratio, ratio of arterial oxygen partial pressure (PaO_2) to inspired oxygen fraction (FiO_2); CRP, C-reactive protein; WBC, white blood cell count; APACHE IV, Acute Physiology and Chronic Health Evaluation IV. Group 1 denotes oXiris and group 0 denotes conventional CVVHD (continuous venovenous hemodialysis).
