# Supplementary material for: oXiris-Based Blood Purification Efficacy in Adults with Septic Shock and Acute Kidney Injury: The PURE Multicenter Pilot Randomized Clinical Trial
Source: J Clin Med. 2026 Oct 16;15(20):8028. doi: 10.3390/jcm15208028 (PMC13706304; doi:10.3390/jcm15208028)
Supplement: Supplementary file 1 [file jcm-15-08028-s001.zip › Table_S4_Archived_variance_inflation_factors.pdf]

Table S4. Archived variance inflation factors

Variable	VIF
Group	1.121
Age	1.053
MAP	1.048
P/F ratio	1.101
Lactate	1.063
Creatinine	1.063
Platelets	1.040
CRP	1.049
WBC	1.084
APACHE IV	1.027

Low VIFs describe linear dependence among the model terms; they do not establish adequate model size, calibration, or control of unmeasured differences.

Abbreviations: VIF, variance inflation factor; MAP, mean arterial pressure; P/F ratio, ratio of arterial oxygen partial pressure (PaO_2) to inspired oxygen fraction (FiO_2); CRP, C-reactive protein; WBC, white blood cell count; APACHE IV, Acute Physiology and Chronic Health Evaluation IV.
