# Supplementary material for: oXiris-Based Blood Purification Efficacy in Adults with Septic Shock and Acute Kidney Injury: The PURE Multicenter Pilot Randomized Clinical Trial
Source: J Clin Med. 2026 Oct 16;15(20):8028. doi: 10.3390/jcm15208028 (PMC13706304; doi:10.3390/jcm15208028)
Supplement: Supplementary file 1 [file jcm-15-08028-s001.zip › Table_S5_Archived_full_covariate-adjusted_model.pdf]

Table S5. Archived full covariate-adjusted model

Variable	Adj. Coef. (β)	Adjusted OR	95% CI	p-value
Group (1 vs 0)	−0.495	0.610	0.326 – 1.141	0.122
Age (per year)	0.065	1.067	1.005 – 1.133	0.034
MAP (per mmHg)	0.030	1.031	0.996 – 1.066	0.080
P/F ratio (per unit)	−0.005	0.995	0.991 – 0.999	0.015
Lactate (per mmol/L)	−0.011	0.989	0.877 – 1.115	0.855
Creatinine (per µmol/L)	0.001	1.001	0.997 – 1.005	0.520
Platelets (per $\times 10^9$/L)	0.010	1.010	0.997 – 1.022	0.124
CRP (per mg/L)	−0.004	0.996	0.969 – 1.024	0.800
WBC (per $\times 10^9$/L)	−0.057	0.945	0.871 – 1.025	0.172
APACHE IV (per point)	−0.001	0.999	0.992 – 1.007	0.881

Archived full model: 202 analyzed participants and 87 deaths. Treatment OR 0.610 (95% CI 0.326–1.141; p = 0.122). No participant-level refit or missing-data reconstruction was performed. The source reports intercept −4.294 (95% CI −10.734 to 2.147; p = 0.191). See Note S2 for the unresolved creatinine-validation and imputation issues. Asterisks denoting significance were removed; numerical p-values are retained.

Abbreviations: Adj., adjusted; Coef., coefficient; β, logistic-regression coefficient on the log-odds scale; OR, odds ratio; CI, confidence interval; MAP, mean arterial pressure; P/F ratio, ratio of arterial oxygen partial pressure (PaO_2) to inspired oxygen fraction (FiO_2); CRP, C-reactive protein; WBC, white blood cell count; APACHE IV, Acute Physiology and Chronic Health Evaluation IV. Group 1 denotes oXiris and group 0 denotes conventional CVVHD (continuous venovenous hemodialysis).
