# Supplementary material for: oXiris-Based Blood Purification Efficacy in Adults with Septic Shock and Acute Kidney Injury: The PURE Multicenter Pilot Randomized Clinical Trial
Source: J Clin Med. 2026 Oct 16;15(20):8028. doi: 10.3390/jcm15208028 (PMC13706304; doi:10.3390/jcm15208028)
Supplement: Supplementary file 1 [file jcm-15-08028-s001.zip › Table_S6_Archived_classification_table.pdf]

Table S6. Archived classification table

	Predicted: Alive	Predicted: Dead
Observed: Alive (n=115)	90	25
Observed: Dead (n=87)	49	38

Threshold 0.5. Source counts correspond to 128/202 correctly classified, sensitivity 38/87, and specificity 90/115. This is an in-sample diagnostic, not validated clinical prediction.

Abbreviations: n, number of participants.
