# Supplementary material for: oXiris-Based Blood Purification Efficacy in Adults with Septic Shock and Acute Kidney Injury: The PURE Multicenter Pilot Randomized Clinical Trial
Source: J Clin Med. 2026 Oct 16;15(20):8028. doi: 10.3390/jcm15208028 (PMC13706304; doi:10.3390/jcm15208028)
Supplement: Supplementary file 1 [file jcm-15-08028-s001.zip › Table_S7_Archived_Box–Tidwell_output.pdf]

Table S7. Archived Box–Tidwell output

Variable	Box–Tidwell p-value
Age	0.589
MAP	0.487
P/F ratio	0.188
Lactate	0.543
Platelets	0.231
CRP	0.352
WBC	0.709
APACHE IV	0.904

Only eight continuous-variable tests were reported. The source states that creatinine was omitted because of an implausible negative value; its origin and model handling are unresolved. Nonsignificant tests do not establish that assumptions are satisfied. See Note S2.

Abbreviations: MAP, mean arterial pressure; P/F ratio, ratio of arterial oxygen partial pressure (PaO_2) to inspired oxygen fraction (FiO_2); CRP, C-reactive protein; WBC, white blood cell count; APACHE IV, Acute Physiology and Chronic Health Evaluation IV.
