# Supplementary material for: oXiris-Based Blood Purification Efficacy in Adults with Septic Shock and Acute Kidney Injury: The PURE Multicenter Pilot Randomized Clinical Trial
Source: J Clin Med. 2026 Oct 16;15(20):8028. doi: 10.3390/jcm15208028 (PMC13706304; doi:10.3390/jcm15208028)
Supplement: Supplementary file 1 [file jcm-15-08028-s001.zip › Table_S8_Unadjusted_Bayesian_Results.pdf]

Table S8. Reproducible post hoc unadjusted Bayesian results

Analysis	OR	95% interval	P(OR < 1)
Approximate indirect prior, 75% precision	0.800	0.363–1.761 (prior interval)	Not a trial result
PURE unadjusted likelihood	0.719	0.411–1.258 (CI)	87.6% with flat log-OR prior
Posterior, 75% reference precision	0.745	0.472–1.176 (CrI)	89.7%

Approximate normal–normal calculation from reported mortality counts. The prior is indirect and post hoc. The unmatched adjusted Bayesian output is omitted and explained in Note S3; it is not silently replaced.

Abbreviations: OR, odds ratio; CI, confidence interval; CrI, credible interval; P(OR < 1), probability that the mortality odds ratio for oXiris relative to control is below 1 under the stated model.
