# Supplementary material for: oXiris-Based Blood Purification Efficacy in Adults with Septic Shock and Acute Kidney Injury: The PURE Multicenter Pilot Randomized Clinical Trial
Source: J Clin Med. 2026 Oct 16;15(20):8028. doi: 10.3390/jcm15208028 (PMC13706304; doi:10.3390/jcm15208028)
Supplement: Supplementary file 1 [file jcm-15-08028-s001.zip › Table_S9_Bayesian_Prior_Sensitivity.pdf]

Table S9. Unadjusted Bayesian prior-sensitivity analysis

Retained prior precision	Posterior OR	95% CrI	P(OR < 1)
0%	0.719	0.411–1.258	87.6%
25%	0.730	0.435–1.225	88.3%
50%	0.739	0.455–1.199	89.0%
75% (reference)	0.745	0.472–1.176	89.7%
100%	0.751	0.487–1.157	90.3%

The 75% scenario is a post hoc reference, not a prespecified success criterion. At 0%, the prior is flat on the log-odds scale. Values are calculated by Code S1 from the retained unadjusted likelihood.

Abbreviations: OR, odds ratio; CrI, credible interval; P(OR < 1), posterior probability that the mortality odds ratio for oXiris relative to control is below 1 under the stated model.
